# Ground-Level Geriatric Falls: A Not-So-Minor Mechanism of Injury

**DOI:** 10.1155/2014/164632

**Published:** 2014-11-06

**Authors:** Simon Parker, Arash Afsharpad

**Affiliations:** ^1^North Central Thames Foundation School, UCL Medical School, Royal Free Hospital, Room GF/664, Rowland Hill Street, London NW3 2PF, UK; ^2^Trauma & Orthopaedics, Barnet Hospital, Wellhouse Lane, Barnet, Hertfordshire EN5 3DJ, UK

## Abstract

*Introduction*. Ground-level falls are typically regarded as a minor mechanism of injury that do not necessitate trauma team activation; however, they represent a significant proportion of hospitalised trauma and can result in multisystem injury. *Case Presentation*. A 79-year-old nursing home resident was brought to the emergency department following an unwitnessed fall. She suffered dementia and had a seizure in the department resulting in a reduced GCS, making history and examination difficult. She was diagnosed with a right proximal humerus fracture and admitted under joint orthopedic and medical care. Following orthopedic review, further X-rays were requested which showed bilateral neck of femur fractures. The following day she had bilateral hip hemiarthroplasties and K-wire stabilisation of the right shoulder. Several days later, when cognition had improved, she was noted to be avoiding use of the left arm and was found to also have a left proximal humerus fracture which was managed conservatively. *Conclusion*. Trauma patients with reduced cognitive function should undergo full ATLS assessment, and a prospective trial is required to see if age should be incorporated as a criteria for trauma team activation. More liberal use of advanced imaging such as a full body CT-scan may be beneficial.

## 1. Introduction

This case report highlights the importance of carrying out a full trauma assessment in geriatric patients with a severity of cognitive impairment that prevents them from communicating the mechanism of their injury and symptoms. It also highlights that reliance on a limited, unwitnessed account to guide your assessment and management can be extremely detrimental to the patient and can result in missed injuries. With the progressively increasing pressure on A&E departments to see and refer patients quickly, this is an all too often occurrence and demonstrates the crucial need of a care pathway for this group of patients to prevent similar incidents in future.

Ground level falls in geriatric patients are a common presentation and represent a significant proportion of hospitalised trauma cases, a trend which is set to continue in the presence of an ever aging population [1]. This type of accident is traditionally considered a minor mechanism of injury and deemed not to necessitate transfer to a trauma centre. However, many of these patients can sustain severe multisystem injuries, even if initially appearing stable [2, 3]. The combination of a greater likelihood of extensive medical comorbidities and a difficult, less-thorough assessment means that elderly trauma victims have significantly worse outcomes compared to their younger counterparts [4].

We discuss these issues in the context of an elderly nursing home resident who had multiple fractures missed following initial assessment for an unwitnessed fall.

## 2. Case Presentation

A 79-year-old nursing home resident was brought in by ambulance to her local A&E after being found on the floor in her bedroom following an “unwitnessed fall.” The accident had occurred within the preceding hour, as she had received her morning medication without concern.

On arrival she was maintaining her own airway and speaking in full sentences, with a respiratory rate of 20, oxygen saturations of 98% on room air, and a clear chest on auscultation. Her C-spine had not been immobilised as it was not deemed necessary following paramedic assessment. She was warm and well perfused with no signs of open trauma or external hemorrhage. Her heart rate was 97 beats per minute and blood pressure was 163/98 mmHg. She had slightly clammy palms but was apyrexial on admission. She was also fully alert but disorientated, with a Glasgow Coma Score of 14 and pupils that were equal and reactive to light. ECG showed regular, sinus rhythm.

The only obvious injuries on initial inspection included bruising around the left eye and right shoulder. Exposure of the patient to assess for other injuries was interrupted, however, as she proceeded to have a tonic-clonic seizure in A&E. This lasted 2-3 minutes and self-terminated but left her with a GCS of 13/15 (E3, V4, M6) in the postictal period, making further history and examination difficult.

Discussion with her nursing home provided limited information. Her past medical history included Parkinson's disease, dementia, hypertension, and asthma, and over the last 3 days they described an increasing level of confusion and poor communication relative to her normal state, but little else of note. They were unable to provide any information about her fall, simply saying she had been found on the floor of her bedroom by a nurse.

Following the postictal period she was further assessed but no other injuries were identified. Her abbreviated mental test score at this time was 0/10 with no baseline level of cognition with which to compare.

**Figure 1:**
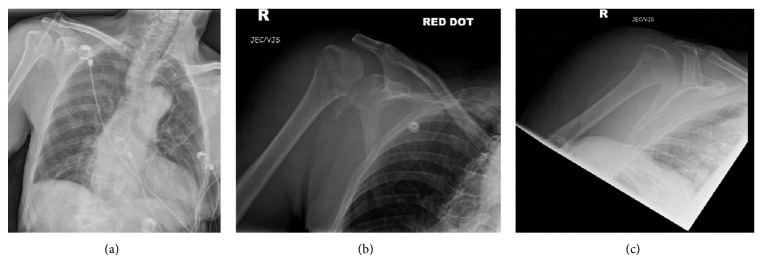
AP chest (a) and right shoulder AP (b) and Y-view (c) radiographs in A&E.

She had a head CT, which excluded acute intracranial pathology, and chest and right shoulder radiographs (Figure 1) which identified a fracture dislocation of the shoulder. She was then referred to both medical and orthopedic specialties for admission.

**Figure 2:**
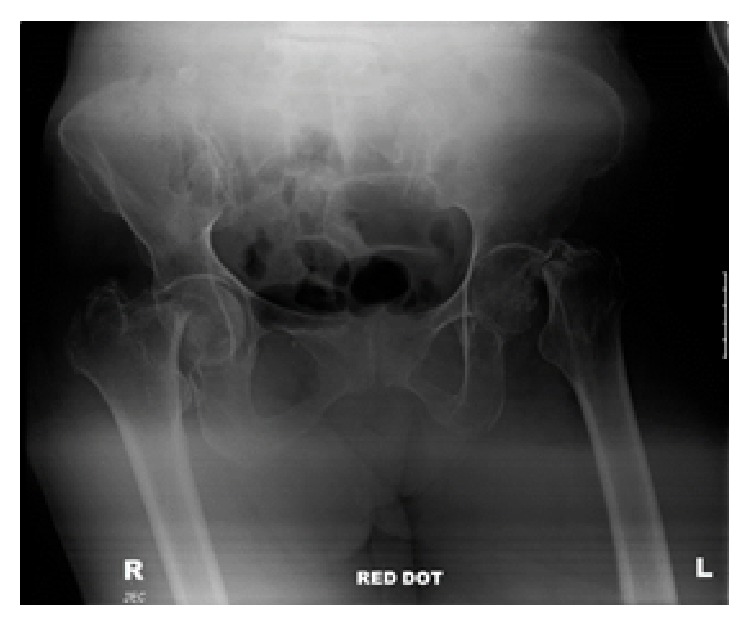
AP X-ray pelvis showing bilateral displaced intracapsular neck of femur fractures.

Orthopedic review identified that both legs were held in an awkward posture and that the patient was not actively moving either side, despite having previously been able to walk small distances assisted with a Zimmer frame. There was a reasonable degree of passive movement at each hip joint, associated with a small amount of pain generally, ascertained by facial grimacing rather than direct communication. Examination of the other extremities was unremarkable. On this basis, further pelvic and hip radiographs were requested (Figure 2).

**Figure 3:**
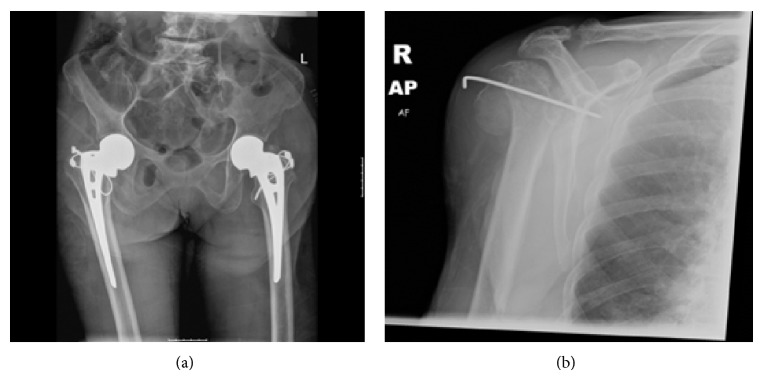
Postoperative radiographs demonstrating (a) bilateral hemiarthoplasties and (b) K-wire stabilisation of the right shoulder.

Pelvic radiographs clearly identified bilateral intracapsular neck of femur fractures, and the patient was taken to theatre the following day for bilateral hemiarthroplasties and manipulation and K-wire stabilisation of her right dislocated shoulder (Figure 3). Intraoperatively, the anesthetist raised concerns over the patient's oxygen saturation and blood pressure, and it was noted that both neck of femur fractures were associated with lesser trochanter extension. The decision was taken that the best option for this patient would be for a fast procedure with reduced anesthetic time, and, therefore, an uncemented Austin Moore prosthesis was used with additional cable stabilisation. Although NICE guidance now recommends the avoidance of such prostheses, in this clinical setting the long term outcome of the prosthesis was deemed less important than a fast and effective operation due to the patient's comorbidities.

**Figure 4:**
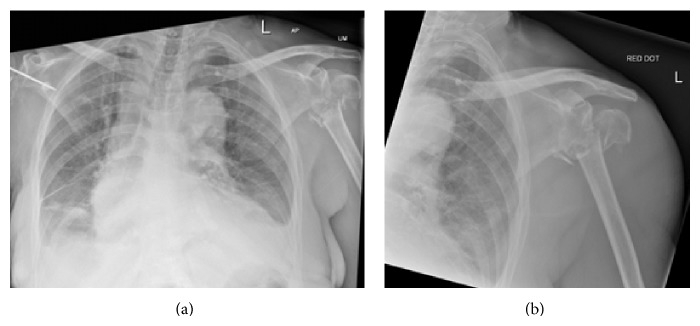
AP radiographs of the left shoulder showing a proximal humeral fracture (a, b).

She continued to be seen by both medical and orthopedic teams on the ward postoperatively, and, although remaining stable, she still appeared drowsy and unresponsive relative to her normal state, making further history and examination an ongoing struggle. With regular review, however, it was noted that she rarely used her left arm, still automatically favouring the right for light manual tasks, despite the history of trauma to the right shoulder. She also developed bruising around the left shoulder and upper arm over the next few days, and this prompted further imaging, which revealed a left proximal humerus fracture (Figure 4). It is most likely that this was a missed injury that had not been picked up on admission, in part, due the fact that the initial AP chest radiograph did not show the left shoulder and also due to the difficulties of assessment.

Given the anaesthetic difficulties in the previous operation, the decision was taken to avoid further surgery and manage this fracture conservatively with a collar and cuff. Our patient went on to have a turbulent admission, suffering several further seizures and requiring antibiotics for a chest infection. However, despite these obstacles she is now well enough to be discharged back to her nursing home.

## 3. Discussion and Conclusions

Our case demonstrates the severity of injury that can be sustained following what would traditionally be considered a minor mechanism. Although no history about the fall was obtainable in this case, the authors suspect that it may have in fact been related to a seizure, rather than a simple fall, given the further seizures in hospital, the severity of injury, and the nature of having a posterior fracture dislocation of the right shoulder, which is more commonly seen following seizure activity.

These severe injuries corroborate with the current change in attitude towards low impact trauma, with several studies demonstrating the significant morbidity and mortality associated with such cases [1, 2, 4]. This is particularly true of geriatric trauma compared to the younger population. The most recent data recommends a cutoff of 70 years at which to consider a patient with trauma to be elderly and, therefore, at an increased risk of mortality for a given injury severity score [5]. Velmahos et al. determined that being over 55 years is an independent risk factor for significant injury in patients having a fall from a low height [6]. However, those that survive the initial burden of the trauma will often achieve some level of functional outcome [7].

Current ATLS teaching recommends transport to a trauma centre for cases involving patients over the age of 55 [8]. However, this calls into question how we determine what a “trauma” case is. Age is not currently treated as an independent factor in the calculation of injury severity, and, therefore, where a hospital or ambulance service utilises scores such as the injury severity score (ISS) or Abbreviated Injury Scale (AIS) as its determinant of trauma activation, the recognised issue of increased mortality rates in the elderly population goes unaccounted for, with patients being transferred to the nearest hospital rather than a trauma centre.

Spaniolas et al. carried out the largest series of trauma patients with ground level falls to date, incorporating 57,302 patients and noting a significant incidence of severely injured elderly patients compared with the nonelderly (11.5% versus 9%, *P* < 0.0001), as well as a mortality rate of 4.4% compared with 1.6%, respectively (*P* < 0.0001). Surprisingly, however, despite the greater average injury severity and worse mortality rates, elderly patients were less likely to be admitted to the trauma service or to receive urgent or emergency surgical interventions [1].

A contributing factor that makes the accurate assessment of the older patient difficult is the lack of available history that often accompanies them. This was certainly true of our case, where the trauma was unwitnessed; the patient had a history of dementia, and shortly into the assessment was left with a GCS of 13/15 following a seizure. There is also the well-recognised fact that elderly patients with a history of dementia tend to report less pain for the same severity of injury than their younger counterparts [9]. The inability of these populations to communicate pain and discomfort is a major barrier to adequate assessment and diagnosis, and, therefore, this alone should mandate a full trauma assessment to avoid missing injuries, which account for a significant healthcare burden [10]. An extended clinical examination should include observation of patient behaviours such as facial expression and body movements as indicators of pain or even the use of behavioural pain assessment tools [11]. It was some 5 days following initial presentation before it became apparent that our patient was avoiding the use of her left arm. During that time we had no verbal communication from her that she may been in pain.

Recent studies have also suggested more liberal use of further imaging to overcome the problem of missed injuries. Dwyer et al. demonstrated that the routine use of a full body CT, or “PAN-SCAN,” may be beneficial in elderly victims of low velocity trauma, by providing evidence of its increasing use and association with decreasing mortality rates over recent years [12]. Other independent associations with PAN-SCAN diagnostic imaging are reduced ITU stay and overall hospital length of stay, the obvious implication being that this would offset the higher initial costs of the investigation.

## Key Messages

(1) Latest research tells us that age should be incorporated into local trauma team activation criteria, but a prospective evaluation of this is still needed [4, 13, 14]. An important factor to address in such a study would be cost, as trauma team activation is an expensive endeavour, particularly when dealing with elderly trauma victims that are already recognised as being associated with an increased cost compared to younger counterparts [15, 16].

(2) Trauma patients with impaired cognitive function should undergo full ATLS assessment to determine level of injury. We should be actively searching for evidence and cause of pain and may benefit from utilisation of additional behavioural pain assessment tools [11].

(3) More liberal use of advanced imaging, such as the full body CT, may help to overcome some of the difficulties associated with the geriatric trauma assessment and reduce rates of missed injuries [12].

Older adults account for some of the most complex and demanding trauma victims we see, in both low and high velocity injuries. They offer unique physiological and structural differences that leave them at an increased risk of mortality; however, those that survive the initial burden will often go on to achieve a functional outcome. They deserve to be triaged, evaluated, and treated with this consideration in mind.
